# Socioeconomic and Demographic Contexts of Brazilian and International University Students Regarding the Use of Dental Services

**DOI:** 10.1155/2022/9362257

**Published:** 2022-03-31

**Authors:** Cosmo Helder Ferreira da Silva, Edmara Chaves Costa, Ana Caroline Rocha de Melo Leite, Vânia Barbosa do Nascimento

**Affiliations:** ^1^University Center Faculty of Medicine of ABC, Graduate Program in Health Sciences, São Paulo, Brazil; ^2^University of International Integration of Afro-Brazilian Lusophony, Nursing Graduate Program, Redenção, Brazil

## Abstract

This article characterizes the different socioeconomic and demographic contexts regarding the use and access to dental services by Brazilian and African students at a Brazilian university of international nature. This is a cross-sectional, analytical, observational study with a quantitative approach, with data produced by 350 students from a public university in the state of Ceará. Sociodemographic and economic factors, participation in educational activities, self-perception of oral health, and use of dental services by academics were analyzed. The results obtained indicated that of the university students participating in the study, 74.0% had already used dental services, of which 57.43% were Brazilian and 42.57% international. There was a significant association between being a Brazilian academic and having already used dental services, having an income less than or equal to the minimum wage, and having used the public dental service. The determination of the prevalence of use of dental services and the different contexts of university students can assist in planning future actions in oral health that prioritize groups of university students with greater difficulties in the use and access of these services.

## 1. Introduction

Despite the advances made in the oral health care process and its public policies, there are still social inequalities in the use of dental services. In this context, the reduced access and use of oral health services raise the need for effective public policies that provide greater assistance in oral health, focusing on the promotion, recovery, and prevention of pathologies. Such policies can act by implementing educational programs and expanding access and regular use of dental services by the population [1].

In the context of the use of oral health services, it is important to assess, among other factors, those related to the socioeconomic and demographic aspects of the populations. In fact, less economically favored individuals have greater limitations on access and use of dental services. From this perspective, evaluating the use of these services and their main limiting factors is extremely necessary to effectively know the challenge of universal access to health proposed by the unified health system (SUS) [2].

In Brazil, for decades, the use of dental services has focused on children and, subsequently, it has focused on private practice and workers with a formal contract. However, in the 2000s, there was an expansion in access to these services through the implementation of the oral health team (ESB) in the family health strategy (ESF). This strategy aimed, in particular, to guarantee access, overcome social inequalities and meet repressed demand [3].

In this context, despite the need to assess the population's access to dental services and their efficiency, especially after the creation of the ESB, several epidemiological studies follow methodologies recommended by the World Health Organization (WHO) that do not include the ages of 19 to 35 years, an age group that includes most university students [4]. Thus, data related to the use of dental services by university students is still scarce.

In several African countries, access to dental services is limited, and diseases of the oral cavity are often left untreated. The existence of caries disease among populations results not only from biological factors, but mainly from the social inequality into which these individuals are inserted [5, 6]. Despite the relevance of care for oral diseases such as caries and periodontal disease worldwide, mainly because it is a challenge to global public health, oral health in some developing countries is still precarious [7].

Specifically, attention to oral health policies for college students becomes crucial when considering the involvement of this population in oral diseases such as dental caries and periodontal diseases. These oral diseases may affect academic performance, damage family and social relationships, generate unnecessary spending on public services and promote systemic, psychological and social complications [8–10].

Dental caries is one of the main reasons for seeking dental services, in addition to being a serious public health problem in several countries, including Africa. Although the indexes show high tooth loss due to caries, this disease can be easily prevented with health education practices and changes in oral hygiene habits [11].

Thus, knowledge about access to oral health services for university students, as well as the factors that determine this use, is relevant to the formulation of health care policies aimed at reducing the impact of oral health on the quality of life of these students, whether Brazilian or international.

Based on the above, the present study aimed to characterize different socioeconomic and demographic contexts regarding the use and access of dental services by Brazilian and African students at a Brazilian university of international nature.

## 2. Materials and Methods

This is a cross-sectional analytical observational study with a quantitative approach, conducted at the University of International Integration of Afro-Brazilian Lusophony (UNILAB), at the Redenção campus and at the Academic Unit of Acarape, Ceará, Brazil. With 3,995 students enrolled in 2019, UNILAB's specific institutional mission is to train human resources to contribute to the integration between Brazil and the member countries of the Community of Portuguese-Speaking Countries (CPLP), especially African countries. In addition to promoting regional development and cultural, scientific, and educational exchange in the region of the state of Ceará [12].

Redenção is a city located in Maciço de Baturité, 61 km away from the capital of the state of Ceará, Fortaleza, and constituting one of the municipalities of the Serra de Guaramiranga Pole. In 2018, the estimated population was 27,633 inhabitants. Its economy is based on agriculture, with the cultivation of bananas, sugar cane, corn, and beans, as well as cattle, swine, and poultry [13, 14]. The municipality of Acarape is 56 km from Fortaleza, located in the microregion of Baturité, a mesoregion of northern Ceará and comprising the Serra de Guaramiranga Pole. In 2018, its estimated population was 15,399 inhabitants. In the municipality, there are mainly activities focused on livestock, agriculture (cultivation of sugar cane, beans, and others), and the exploration of limestone [13, 15].

The research was carried out from January to July 2019 by a team of three interviewers. Data collection was carried out in the morning and afternoon shifts during working days, at preestablished times. The interviews were conducted individually and in a reserved area on the university campus.

They were invited to participate in the academic study of UNILAB, of different nationalities (Angola, Brazil, Cape Verde, Guinea-Bissau, Mozambique, São Tomé and Príncipe, and East Timor), of the bachelor's courses (Public Administration, Agronomy, Bachelor of Humanities-BHU, Natural Sciences and Mathematics, Social Sciences, Nursing, Computer Engineering, and Energy Engineering) and undergraduate courses (Biological Sciences, Letters–Portuguese Language, Mathematics, Pedagogy, and Chemistry). For the inclusion of participants, the following criteria were instituted: to be properly enrolled in a face-to-face undergraduate course; to be attending any semester. Academics absent from UNILAB on the days of questionnaires and oral exams were excluded from the study.

The sample size was calculated in order to meet both the prevalence and association studies between the independent and dependent variables. The sample size was calculated at 350 students, with a response rate of 100%. For this, the sample size calculation was performed based on finite populations, assuming a 95% confidence level, 5% error, and 50% proportion [16].

Data collection started with the explanation of the project and the application of the free and informed consent term (ICF). After that, a validated questionnaire was applied [11] and adapted by the authors, containing objective questions related to sociodemographic and economic aspects, participation in oral health educational actions, self-perception of oral health, access and types of dental services used.

In the first block, for those scholars who made use of dental services, the variables were described as follows: nationality (Brazilian and international); sex (female and male); age in years (≤25 and >25); place of residence (urban and rural areas); color/race, the race/color white, black, and because they are small groups and not liable to form a specific category, individuals of yellow and indigenous skin color were incorporated to those of brown color, configuring the category “brown, yellow and indigenous” [17]; family income (≤1 minimum wage and >1 minimum wage); participation in educational oral health actions (yes and no); types of dental services used (public and private); last visit to the dentist (<1 year and ≥1 year), and perception of oral health (excellent/good, regular, and bad/very bad).

In the second block, the independent variables were represented by: nationality (Brazilian and international); sex (female and male); age in years (≤25 and >25); place of residence (urban and rural areas); family income (≤1 minimum wage and >1 minimum wage); participation in educational oral health actions (yes and no); and excellent/good self-perception of oral health (yes and no). The dependent variables were represented by: participation in educational oral health actions (yes and no); “excellent/good” self-perception of oral health (yes and no); use of dental services (yes and no); types of dental services used (public and private); and last visit to the dentist (<1 year and ≥1 year).

In the third block, the independent variables were: use of dental services (yes and no) and types of dental services used (public and private). The following were considered as dependent variables: participation in educational oral health actions (yes and no); “excellent/good” self-perception of oral health (yes and no); and last visit to the dentist (<1 year and ≥1 year).

The statistical analysis included the calculation of the measures of absolute and relative frequency. Pearson's chi-square and Fisher's exact tests were used to analyze the association between categorical variables. A significance level of 0.05 was adopted. The analysis was performed using the statistical program Epi InfoTM for Windows, version 7.2, available free of charge from the center of disease control and prevention (CDC).

Data collection was conducted according to the ethical principles contained in Resolution no. 466/12 of the National Health Council [18]. The project was approved by the Ethics Committee of the University Center Faculty of Medicine of ABC (FMABC) according to CAAE: 87168218.3.0000.0082 and opinion no. 2,647,693.

## 3. Results

Of the 350 academics who participated in the study, 74.00% used dental services, 57.43% were Brazilian and 42.57% were international academics, 53.30% were male and 73.04% were 25 years old or less. Of this total, 68.88% of the students lived in an urban area, 53.31% declared themselves to be black, 63.42% had an income less than or equal to 1 minimum wage, and 57.20% had already participated in educational oral health actions. Still, 63.81% of these students used the public service, 63.04% had done it less than 1 year ago, and 47.78% had a “great/good” perception of oral health (see Table 1).

When analyzing the association between nationality and participation in educational oral health actions, there was a significant relationship between being an international academic and not having participated in educational activities in oral health (*p*=0.001). For the relationship between nationality, the use of dental services, and the time of the last visit to the dentist, a significant association was observed between being a Brazilian academic and having used dental services (*p*=0.001) and having sought this type of care for less than 1 year (*p*=0.002) (see Table 2).

Source: authors, 2019. As for the association between sex and participation in educational oral health actions, there was a significant relationship between being a male academic and not having participated in this type of activity (*p*=0.046). Regarding the relationship between sex, perceived “good/good” oral health, and the use of dental services, there was a significant association between being a male academic and not having “great/good” perceived oral health (*p*=0.023) and being a female academic and having used dental services (*p*=0.009).

Regarding the association between age and perceived “great/good” oral health, there was a significant relationship between being an academic over the age of 25 and having “great/good” perceived oral health (*p*=0.012). Regarding age and the use of dental services, there was a significant relationship between being academic aged 25 years or less and having used these services (*p*=0.004).

Regarding the relationship between family income, the use of dental services, and the types of dental services used, there was a significant association between being an academic with an income higher than the minimum wage and not using these services (*p*=0.001) and having an income less than or equal to the minimum wage and having used the public dental service (*p*=0.003). For the place of residence, there was a significant relationship between being an academic who lives in a rural area and have used the public dental service (*p*=0.034) and being an academic who lives in an urban area and have sought this type of care for less than 1 year (*p*=0.034).

As for the association between educational oral health actions and perceived “great/good” oral health, there was a significant relationship between not having participated in these actions and not having “great/good” perceived oral health (*p*=0.003). Regarding participation in these actions, the use of dental services and their types, apprehending a significant relationship between the participants in these actions and the use of dental services, public or private (*p*=0.001), and use of public service (*p*=0.007).

Regarding the relationship between perceived “great/good” oral health and participation in educational oral health actions, there was a significant association between not having a “great/good” perception of oral health and not having participated in this type of activity (*p*=0.005). For the relationship between this perception and the time of the last visit to the dentist, a significant association was observed between having a “great/good” perception of oral health and having sought this type of care for 1 year or more (*p*=0.008).

With regard to the association between the use of dental services and participation in oral health educational actions, there was a significant relationship between not using these services and having participated in oral health educational actions (*p*=0.001). As for the relationship between the types of public and private dental services and this participation, a significant association was found between having used the public service and having participated in educational oral health actions (*p*=0.007) (see Table 3).

## 4. Discussion

This study was one of the first to characterize and associate sociodemographic and economic aspects, perception and participation in oral health educational actions, and use of dental services by Brazilian and African students at a Brazilian university of international nature. Its conduction showed, among other results, that a considerable number of participants had used the public service and had a “great/good” perception of oral health. Between the different associations, the research revealed a significant relationship between being an international academic and not having participated in educational oral health actions, as well as being a Brazilian academic and having used dental services and having sought this type of care for less than one year.

Specifically, the characterization and association between the variables mentioned above enabled a more excellent knowledge of the population of students of different nationalities, semesters, and courses at UNILAB, which may better direct the institution of strategies focused on oral health, including the search and use of dental services, with personal, academic, and professional repercussions.

When evaluating the nationality of the participants, the greater participation of international academics was unexpected since UNILAB, although it is an institution of higher education with an international character, has and receives a greater number of Brazilian students in relation to other nationalities. Similar to this result, the greater number of male academics in the present study was surprising since, according to UNILAB data, there is a predominance of female students, especially those aged 25 years or less. With regard to the predominance of participants aged 25 years or less, this finding corroborates the fact that, at the university, more than 50% of students, of both sexes, are in this age group. Regarding the largest number of academics who declared themselves black, although this race is the second most abundant among university graduates, this finding may be a reflection of the significant presence of Africans at UNILAB, as well as result from one of its selection processes, which specifically includes quilombola students [19].

As for household income, the highest number of academics with an income less than or equal to the minimum wage may result from the law's implementation at 12,711/2012, which determined that 50% of places at universities and federal institutions would be for students in full arising public high schools, with a gross family income of 1.5 minimum wages or less [20]. For the largest number of participants living in the urban area, this data can be justified based on the fact that living in the countryside, by significantly increasing the likelihood of an individual being relatively low income [21], can contribute to a lower level of education and more difficult access to university.

According to Neves et al. [22], socioeconomic inequalities directly affect health. Specifically, for the use of dental services, socioeconomic and demographic factors can interfere with usage patterns. In fact, individuals with better socioeconomic conditions may have easier access to health care than people with low incomes. This social inequality in access tends to be greater in countries with a private health system, where the population has to pay for health care, either through plans and insurance or direct payment to the provider, than in countries with a universal system [23].

In this study, the economic factor did not seem to influence the use of dental services by academics, since many of those who had used this type of service had a low income. In particular, the use of this service by Brazilian academics, especially for less than 1 year, may be linked to greater awareness of the importance of oral health and/or the presence of a carious process since, according to the Oral Health Project 2010 [24], the number of cariesfree individuals decreases with increasing age. The project, by mentioning an average of decayed teeth among Brazilian adolescents of 4.25 teeth and a DMFT index of 16.75 among adults aged 35 to 44 years, reinforces the probability of an increase in carious lesions with age, which can support the search for dental care by academics aged 25 years or less, participating in this research. Other studies have also shown a higher frequency of demand for dental services among younger students [25,26].

In this context, it is worth mentioning that oral health care is relevant in the lives of young people. This care can have a direct influence on the self-esteem and socialization of these individuals. In particular, for teenagers, youth people, and adults. Among the oral problems that can interfere with your quality of life, we highlight untreated caries, severe malocclusions, pain, tooth loss, and gum bleeding [1, 27, 28].

In line with the findings observed here, there was a higher prevalence of the use of dental services in the population of Brazilian students in relation to international students [11, 29]. Considering these results, it is possible that the scarcity of oral health professionals, inadequate infrastructure, and the lack of access to dental services in the country of origin, as well as the nonparticipation in educational health actions, may justify the lower use by international scholars of Brazilian dental services [30]. Notably, the noninvolvement in educational activities related to oral health by the international student was evidenced here by the association between being an international academic and not having participated in educational activities in oral health.

With regard to the use of dental services, according to sex, although the greatest amount of use of these services was found among male academics, which may result from their greater number among the participants in this study, the significant difference between these variables only occurred when it involved the female gender. This result, in addition to meeting many published studies [1, 31–33], can be understandable, considering that men have more difficulty in adopting healthy behaviors in relation to women [34]. It is also possible that this phenomenon occurred due to the nonparticipation of the male sex in oral health educational actions, which contributed to the association, observed here, between being a male academic and not having participated in this type of activity.

When assessing the place of residence among those who had done the most or made the most use of dental services, the greater use by those who lived in urban areas, especially less than 1 year ago, was not surprising. Indeed, the literature reports that, in general, there is less use of all types of health services in the rural area when compared to the urban area, as a consequence of the disparities in the offer of these services between these areas [35]. As for use in a period of less than 1 year, both by residents in urban areas and by participants in general, it can be justified based on the recommendations of the American Dental Association. According to her, generally, adults should make an annual visit to the dentist [36].

Also, considering the home location, the relationship observed between being an academic resident in a rural area and having used the public dental service highlights the remarkable dependence of the rural Brazilian population on public health services and reduced adherence to health plans [37]. This use may have been corroborated by the increase in the supply of health services by SUS [38]. Despite the increase in the services offered, access is still limited to the inhabitants of the rural areas due to their geographic conditions, uneven distribution of professionals, and deficiencies in the network of health units. In addition to these factors, they also contribute to the difficulty of accessing the preferred location of the reference network in the urban area [37].

As for the greater use of dental services by academics who declared it to be black, it can be assumed that this phenomenon occurred because they make up most of the sample of this research. It is possible that this increased use derives from an inadequate oral health condition. In fact, the literature mentions a relationship between the highest prevalence and risk of caries, the worst socioeconomic conditions, and the greatest need for dental treatment in the black population [10]. In this context, race/color is considered one of the limiting factors in the use and access of dental services [39].

The greater use of dental services by students with an income equal to or less than the minimum wage, similar to race/color, in this data may result from the greater number of these students among the participants. It is also possible that these academics, having already participated in educational oral health actions, were aware of the importance of periodic visits to the dental surgeon. This possibility is reinforced by the fact that countless students have already participated in oral health education activities and have already used the public dental service [40, 41].

This assumption is also corroborated by the association observed here: academics participating in oral health education actions using dental services, particularly the public, as well as the relationship between being an academic and having used public service and having participated in actions on oral health education. The purpose of health education includes, in addition to knowledge and understanding of oral health conditions, the influence on oral hygiene habits and, consequently, the search for dental services [42]. However, it is possible that the individual did not seek dental care but participated in educational activities on oral health [43], a phenomenon that occurred in the present study. In fact, educational health actions can take place in different spaces, including the school and home environment, without being restricted to basic health units [44–46]. As for the use of public service, it may be a reflection of the participants' low family income [47].

Considering family income, the relationship between income greater than the minimum wage and the nonuse of dental services was unexpected. It is possible that this event results from lower awareness of these students about the importance of oral health, which can be emphasized by the considerable number of academics who had not participated in educational activities on oral health. It can also be assumed that this higher income arises from paid work activities that, associated with academic life, restrict the student's dedication to health care and, consequently, provide fewer opportunities for searching for and using dental services. However, this result needs to be further investigated.

When evaluating perceived oral health, considered an important health indicator, which summarizes the objective and subjective health situation, values, and cultural expectations, the study showed a reasonable number of academics who considered it to be “great/good,” in addition to the significant association between being an academic who had this perception and having not sought dental care for more than a year. This result is different from the literature, which reports that there is a relationship between the perception of oral health and the visit to the dentist [1, 48, 49].

Still in this context, the study showed an association between being a male academic and not having “great/good” perception of oral health. This data may be a reflection of the lesser care given to males to their health, which can be evidenced by the fact that men have greater difficulty in adhering to healthy behaviors and seeking prevention services [33]. For the relationship between being an academic over the age of 25 and having “great/good” perception of oral health, this association can be justified if considered that, in general, greater maturity implies greater responsibility of the individual in different aspects of including systemic and oral health.

In this sense, in addition to the factors age, sex, income, and education, the contribution of the social environment plays an important role in the perception of oral health. Indeed, the social environment, by involving living and working conditions, qualifies, in a different way, the way in which people think, feel, and act, regarding their individual health [15].

In addition to sex and age, a significant relationship was observed between being an academic and not having participated in educational health actions and not having a “great/good” perception of oral health. Specifically, for this finding, its justification may be based on the fact that participation in health education actions, in addition to increasing the chances of using dental services, as already reported, may imply more knowledge, awareness of the conditions of oral health and a better perception of this condition. This assumption can also support the association between being an academic and not having a “great/good” perception of oral health and not having participated in educational activities related to oral health.

As limitations of the study, it can be mentioned that the reduced access to students of UNILAB's evening courses was mentioned since the collection was carried out in the morning and afternoon periods, as well as the impossibility of carrying out the dental evaluation due to the lack of structure and the high number of students.

Despite the limitations, the research revealed the need to conduct educational activities, mainly aimed at the African public residing in the vicinity of the university, seeking, among other aspects, to encourage the search for dental care and the improvement of oral health. It is also necessary to improve public dental services due to the role they provide to the study population and, possibly, to other community members, in addition to offering structure and qualified professionals to carry out educational activities. It is also recommended to conduct research aimed at the dental evaluation of participants to associate it with the services used by the public and their oral and systemic repercussions.

## 5. Conclusion

From the results obtained, it can be concluded that, despite the low family income, the students used dental services, participated in educational activities on oral health, used public service, and had a great/good perception of oral health. Specifically, international students did not seem to participate in oral health educational activities, and Brazilians sought dental care within a reasonable time.

## Figures and Tables

**Table 1:** Description of sociodemographic, economic, and oral health-related aspects of only Brazilian and international academics who used dental services. Redenção e Acarape, CE, Brazil, 2019.

Variables	N (*N* = 257)^*∗*^	%
Nationality		
Brazilians	148	57.43
International	109	42.57
Sex		
Female	120	46.60
Male	137	53.30
Age (years)		
≤25	187	73.04
>25	70	26.96
Place of residence		
Urban area	177	68.88
Countryside	80	31.12
Color/race		
White	13	5.06
Black	137	53.31
Brown, yellow, and indigenous	107	41.63
Family income^a^		
≤1 MW	163	63.42
>1 MW	94	36.58
Participation in educational actions		
Oral health		
Yes	147	57.20
No	110	42.80
Type of dental service used		
Public	164	63.81
Private	93	36.19
Last visit to the dentist		
<1 year	162	63.04
≥1–2 years	95	36.96
Perception of oral health		
Great/good	122	47.78
Regular	119	46.30
Bad/terrible	16	6.22

^a^MW–minimum wage (2019) which is R$ 998.00. ^*∗*^ Variable values based on the sample of 257 participants who used dental services.

**Table 2:** Association between sociodemographic and economic factors, participation in educational activities, and perception of health and oral health by Brazilian and international academics. Redenção e Acarape, CE, Brazil, 2019.

Variables	Participation in educational actions oral health *n* (%)		“Great/good” perception of oral health *n* (%)		Use of dental services *n* (%)		Types of dental services^d^*n* (%)		Last visit to the dentist^d^*n* (%)		*p* value
	Sim	Não	Sim	Não	Sim	Não	Publ.^b^	Priv.^c^	<1 ano	≥1 ano	
Nationality											
Brazilians	98 (66.22)	50 (33.78)	74 (50.00)	74 (50.00)	148^2^ (100.00)	—	94 (63.51)	54 (36.49)	1053*∗* (70.95)	43 (29.05)	*p* < 0.05
International	73 (63.86)	129^1^ (36.14)	94 (46.53)	108 (53.47)	109 (53.96)	93 (46.04)	70 (64.22)	39 (35.78)	57 (52.29)	52 (47.71)	
Sex											
Female	82 (55.03)	67 (44.97)	82 (55.03)	67 (44.97)	120^6^ (80.54)	29 (19.46)	76 (63.33)	44 (36.67)	78 (65.00)	42 (35.00)	*p* < 0.05
Male	89 (44.28)	112^4^ (55.72)	86 (42.79)	115^5^ (57.21)	137 (68.16)	64 (31.84)	88 (64.23)	49 (35.77)	84 (61.31)	53 (38.69)	
Age											
≤25 years	122 (50.83)	118 (49.17)	126 (52.50)	114 (47.50)	187^8^*∗* (77.92)	53 (22.08)	118 (63.10)	69 (36.90)	114 (60.96)	73 (39.04)	*p* < 0.05
>25 years	49 (44.55)	61 (55.45)	42^7^*∗* (38.18)	68 (61.82)	70 (63.64)	40 (36.36)	46 (65.71)	24 (34.29)	48 (68.57)	22 (31.43)	
Income^a^											
≤1 MW	114 (47.11)	128 (52.89)	112 (46.28)	130 (53.89)	163 (67.36)	79 (32.64)	115^10^*∗* (70.55)	48 (29.45)	107 (65.64)	56 (34.36)	*p* < 0.05
>1 MW	57 (52.78)	51 (47.22)	56 (51.85)	52 (48.15)	94 (87.04)	14^9^*∗* (12.96)	49 (52.13)	45 (47.87)	55 (58.51)	39 (41.49)	
Place of residence											
Countryside	50 (50.00)	50 (50.00)	47 (47.00) (53.00)	53 (53.00)	80 (80.00) (20.00)	20 (20.00)	58^11^^*∗∗*^ (72.50)	22 (27.50)	58 (72.50)	22 (27.50)	*p* < 0.05
Urban area	121 (48.40)	129 (51.60)	121 (48.40)	129 (51.60)	177 (70.80)	73 (29.20)	106 (59.89)	71 (40.11)	104^12^ (58.76)	73 (41.24)	
Participation in educational actions of oral health											
	—	—	95 (55.56)	76 (44.44)	147^14^ (85.96)	24 (14.04)	104^15^ (70.75)	43 (29.25)	98 (66.67)	49 (33.33)	*p* < 0.05
No	—	—	73 (40.78)	106^13^ (59.22)	110 (61.45)	69 (38.55)	60 (54.55)	50 (45.45)	64 (58.18)	46 (41.82)	
“Great/good” perception of oral health											
Yes	95 (56.55)	73 (43.45)	—	—	122 (72.62) (27.38)	46 (27.38)	83 (68.03) (31,97)	39 (31.97)	87 (71.31)	35^17^*∗* (28.69)	*p* < 0.05
No	76 (41.76)	106^16^ (58.24)	—	—	135 (74.18)	47 (25.82)	81 (60.00	54 (40.00)	75 (55.56)	60 (44.44)	

^a^MW-minimum wage (2019) which is R$ 998.00; ^b^Publ.-public; ^c^Priv.-private; *∗* Pearson's chi-square test; ^*∗∗*^*Fisher'*s exact test; ^1^*p*=0.001; ^2^*p*=0.001; ^3^*p*=0.002; ^4^*p*=0.046; ^5^*p*=0.023;^6^*p*=0.009; ^7^*p*=0.012; ^8^*p*=0.004; ^9^*p*=0.001; ^10^*p*=0.003; ^11^*p*=0.034; ^12^*p*=0.034; ^13^*p*=0.003; ^14^*p*=0.001; ^15^*p*=0.007;^16^*p*=0.005; and ^17^*p*=0.008. ^d^ Variable values based on a sample of 257 participants who used dental services.

**Table 3:** Association between types, time and use of dental services, participation in educational activities, and self-perception of health and oral health by Brazilian and international academics. Redenção e Acarape, CE, Brazil, 2019.

Variables	Participation in educational actions of oral health n (%)		“Great/good” perception of hygiene/oral health n (%)		Last visit to the dentist (years) n (%)		*p* value^*∗*^
	Sim	Não	Sim	Não	<1	≥1	
Use of dental services							
Yes	147 (57.20)	110 (42.80)	122 (47.47)	135 (57.20)	162 (63.04)	95 (36.96)	*p* < 0.05
No	24^1^*∗*(25.81)	69 (74.19)	46 (49.46)	47 (50.54)	—	—	
Type of services							
Public	104^2^*∗*(63.41)	60 (36.59)	83 (50.61)	81 (49.39)	102 (62.20)	62 (37.80)	*p* < 0.05
Private	43 (46.24)	50 (53.76)	39 (41.94)	54 (58.06)	60 (64.52)	33 (35.48)	

^*∗*^Pearson's chi-square test; ^1^*p*=0.001 and ^2^*p*=0.007.

## Data Availability

The data used to support the findings of this study are available from the corresponding author upon request.
